# Subacute toxicity evaluations of LPM3480392 in rats, a full µ-opioid receptor biased agonist

**DOI:** 10.3389/fphar.2023.1218380

**Published:** 2023-08-04

**Authors:** Liang Ye, Chunmei Li, Wanglin Jiang, Yifei Yang, Wenyan Wang, Haibo Zhu, Zhengping Hu, Ning Li, Xiaobo Cen, Hongbo Wang, Jingwei Tian

**Affiliations:** ^1^ School of Public Health and Management, Binzhou Medical University, Yantai, Shandong, China; ^2^ Key Laboratory of Molecular Pharmacology and Drug Evaluation, School of Pharmacy, Ministry of Education, Collaborative Innovation Center of Advanced Drug Delivery System and Biotech Drugs in Universities of Shandong, Yantai University, Yantai, Shandong, China; ^3^ School of Pharmacy, Binzhou Medical University, Yantai, China; ^4^ Medicine and Pharmacy Research Center, Binzhou Medical University, Yantai, Shandong, China; ^5^ WestChina-Frontier PharmaTech Co., Ltd., Chengdu, Sichuan, China

**Keywords:** LPM3480392, a full μ-opioid receptor biased agonist, pain, subacute toxicity, rats

## Abstract

Opiates produce analgesia via G-protein signaling, and adverse effects, such as respiratory depression and decreased bowel motility, by β-arrestin pathway. Oliceridine, a G protein-biased MOR agonist, only presents modest safety advantages as compared to other opiates in clinical trials, possibly due to its limited bias. Our previous study shown that LPM3480392, a full MOR biased agonist, is selective for the Gi pathway over the β-arrestin-2. In the present article, we evaluated the subacute toxicity of LPM3480392 in rats. The rats were administered with control article or LPM3480392 0.6, 1.2 or 2.4 mg/kg/day for 4 consecutive weeks followed by a 4-week recovery phase. Intravenous infusion was conducted at tail vein at 0.2, 0.4 or 0.8 mg/kg/day with a dosing volume of 10 mL/kg and 5 min/rat/dose, three times a day with an interval of approximately 4 h. The concomitant toxicokinetics study was conducted. Two unscheduled rats at 2.4 mg/kg/day died with no clear cause. For the scheduled necropsy, the major effects were associated with the MOR agonist-related pharmacodynamic properties of LPM3480392 (e.g., increased activity, increased muscle tone; decreased food consumption and body weight gain; and clinical chemistry changes related with decreased food consumption) in three LPM3480392 groups. In addition, LPM3480392 at 2.4 mg/kg/day also induced deep respiration and histopathology changes in testis and epididymis in sporadic individual rats. However, different from other opiates, LPM3480392 presents weak/no immunosuppression and the decreased adrenal gland weight, which may be due to LPM3480392’ full MOR bias. At the end of recovery phase, all findings were recovered to some extent or completely. In the toxicokinetics study, the dose-dependent elevation of drug exposure was observed, which partly explained the toxicity of high dose. In summary, LPM3480392 has exhibited good safety characteristics in this subacute toxicity study in rats.

## 1 Introduction

Acute postoperative pain is experienced in more than 80% of all surgical patients, with the moderate to severe intensity in at least 75% of these patients (Gan et al., 2014; Gan, 2017). Opiates, especially µ-opioid receptor (MOR) agonists such as morphine and fentanyl, are first-line treatment options for the treatment of moderate to severe pain (Bostrom et al., 1997; Tan and Habib, 2021). Although opiates are effective analgesics, they also induced severe adverse effects (SAE) such as addiction, respiratory suppression, and constipation, thereby limiting their clinical utilization (Del Vecchio et al., 2017; Zhuang et al., 2022). Opiate-induced side effects result in significant morbidity and mortality. For instance, opiate-induced respiratory depression is associated with 15,000 deaths per year, the majority of which are related to illicit and recreational opiate use (Mattson et al., 2021; Tan and Habib, 2021; Zhuang et al., 2022).

As a G protein-coupled receptor (GPCR), MOR has been revealed as the main receptor for both the analgesic and adverse effects of morphine (Matthes et al., 1996). Signaling of MOR is primarily transduced through Gi to inhibit cAMP production. MOR can also signal through β-arrestin upon the receptor activation (Zhuang et al., 2022). The activation of the Gi signaling is responsible for analgesia, while the activation of the β-arrestin pathway contributes to unwanted effects of MOR activation such as respiratory depression and constipation (Bohn et al., 1999; Bohn et al., 2000; Raehal et al., 2005). Therefore, by selectively acting on the Gi signaling pathway in preference to the β-arrestin pathway, there is an interesting drug development strategy with preserving the analgesic activity and avoiding unwanted MOR-associated opiate side effects.

Biased agonism refers to the ability of compounds to drive preferred signaling pathways and avoid adverse signaling pathways in a ligand-dependent manner for some GPCRs (Chan et al., 2017; Daugvilaite et al., 2017; Yang et al., 2022). Some studies have indicated that MOR agonists that are functionally selective for the Gi pathway over the β-arrestin-2, which may elicit analgesia while reducing some side-effects of opiates. Several MOR agonists with negligible β-arrestin activities and potentially with better therapeutic windows than morphine and fentanyl have been identified and characterized, including oliceridine (TRV130), PZM21, and SR17018 (DeWire et al., 2013; Manglik et al., 2016; Schmid et al., 2017; Zhuang et al., 2022). Oliceridine (Olinvyk^®^ Trevena, PA, United States) was approved by the US Food and Drug Administration (FDA) for the treatment of moderate to severe pain on 8 Aug 2020 (Goudra, 2022), which is a G protein-biased MOR agonist that preferentially activates the inhibitory Gi signaling pathway over β-arrestin-2 (Chen et al., 2013). However, in phase III clinical trials, TRV130 did not present its superiority to morphine in terms of the respiratory depression in humans (Kliewer et al., 2020). Recently, a question was generated that the degree of bias will impact the separation of analgesia and respiratory depression side effects (Schmid et al., 2017). Therefore, full MOR biased agonists need to be designed for obtaining higher MOR bias with enough safety margin.

In the previous study, we rationally designed and synthesized a series of potent highly biased MOR agonists through the modification and structure-activity relationship study of TRV130 (Yang et al., 2022). Among these compounds, LPM3480392 demonstrated improved *in vitro* biased agonism (EC_50_ = 0.35 nM, E_max_ = 91.4%) without β-arrestin-2 recruitment activity (EC_50_ > 30,000 nM, E_max_ = 1.6%), good brain penetration, a favorable pharmacokinetic profile and produced potent antinociceptive effect with reduced respiratory suppression as compared to TRV130.

Previously, we have completed a series of toxicity studies in order to support the clinical trials in agreement with the 21 CFR Part 58 of US FDA, Good Laboratory Practice (GLP) regulations. Until now, LPM3480392 has completed phase I clinical trials (CTR20210370; CTR20212865) and is currently under phase II clinical trial (CTR20222358) as an analgesic for the treatment of moderate to severe pain. In this article, we present the subacute toxicity study for LPM3480392 in rats.

## 2 Materials and methods

### 2.1 Materials

**FIGURE 1:**
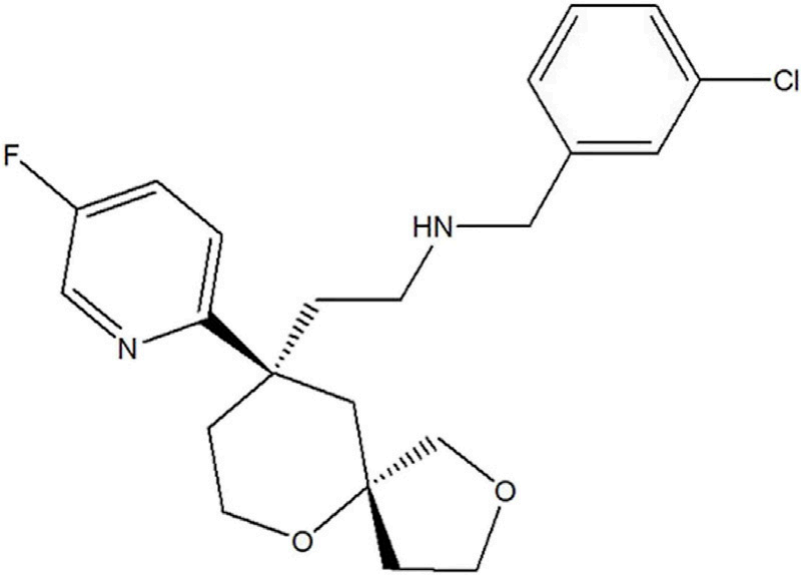
Chemical structure of LPM3480392.

LPM3480392 maleate injection (6.45 mg/5 mL) with a chemical purity of >97.9% was provided by the State Key Laboratory of Long-acting and Targeting Drug Delivery Technologies (Yantai, China) (Figure 1).

### 2.2 Experimental animals

The Sprague Dawley (SD) rats of SPF grade (76 females and 76 males) were from Beijing Vital River Laboratory Animal Co., Ltd. (Production license No.: SCXK (Jing) 016–0011), with the body weight of 132.8–174.6 g and the ages of 6–7 weeks. The rats were supplied with rat/mouse breeding diet *ad libitum* manufactured by Beijing Keao Xieli Feed Co., Ltd (Production License Number: Jingsi license (2018) 06,073). The animals were housed under 12 h light/dark cycles at 21.24°C–25.55°C in 45.23%–72.01% relative humidity environment. All animals had free access to water and food during the experimental periods. This subacute toxicity study has been conducted in WestChina-Frontier PharmaTech Co., Ltd. (WCFP). Animal experiments complied with the relevant regulations in Institutional Animal Care and Use Committee (IACUC) in WCFP.

### 2.3 Study design

The rats were assigned to 4 groups, including control group, and three LPM3480392 injection groups, with 19 rats/sex in each group. The rats were administered with control article (0.9% sodium chloride injection) or LPM3480392 injection 0.6, 1.2 or 2.4 mg/kg/day for 4 consecutive weeks followed by a 4-week recovery phase. Intravenous infusion was conducted at tail vein at 0.2, 0.4 or 0.8 mg/kg with a dosing volume of 10 mL/kg and 5 min/rat/dose using micro-injection pump, three times a day with an interval of approximately 4 h. The first dosing day was defined as Day 1, and the day after the last dosing was defined as Recovery day 1. At 1 day after the last dosing, 10 rats per sex in each group (9 males and 10 females in 2.4 mg/kg/day group) were anesthetized with pentobarbital sodium (i.p., 60 mg/kg) followed by abdominal aorta exsanguination for a gross necropsy based on AVMA Guidelines for the Euthanasia of Animals: 2020 Edition (the American Veterinary Medical Association, 2020). At the end of recovery phase, the 5 animals per sex in each group (5 males and 4 females in 2.4 mg/kg/day group) were sacrificed. For the toxicokinetics study, 4 rats/sex/group were used in three LPM3480392 injection groups.

### 2.4 Preparation and analysis of dose formulation

LPM3480392 injection was diluted with 0.9% NaCl injection to the required concentration under the aseptic condition. The dose formulations were prepared at least once every 8 days. The prepared dose formulations were used within 192 h when stored at 15°C–25°C. Dose formulations in control group and LPM3480392 groups were analyzed for the first and last dosing. The sample (0.100 mL) was collected from the middle layer of control formulation, or LPM3480392 injection dose formulations at each concentration. The absence of LPM3480392 should be confirmed in control formulations. The relative error (RE%) between detected result and nominal value should be within ±10% for LPM3480392 injection dose formulations at different concentrations.

### 2.5 Clinical observation

For all surviving animals, a morning and an afternoon observation every day were conducted for the moribundity and death. Daily observation was carried out once within 1 h after each dosing in dosing phase, and at least once daily in recovery phase, including mental status, food and water consumption, hair, discharge, mortality and other toxic reactions. Detailed observation was performed once every week, including appearance, movement, mental status, gland secretions, skin and mucosa color, respiration, genitals, mortality and other toxic reactions.

### 2.6 Body weight

Body weight was recorded twice a week during the dosing phase and then once a week during the recovery phase. Terminal body weight was recorded before each necropsy for calculating the organ weight ratios.

### 2.7 Food consumption

Food consumption of all surviving animals was recorded twice a week during the dosing phase and then once a week in last 2 weeks of recovery phase.

### 2.8 Ophthalmic examination

The examination was conducted on rats in control and 2.4 mg/kg/day groups at the end of dosing phase (Day 27) and at the end of recovery phase (Recovery day 27). The mydriasis (eye instillation, 1-2 drops/eye) was conducted using Mydrin-P (compound tropicamide eye drops) before examination. Binocular indirect ophthalmoscope was used to examine conjunctiva, cornea, iris, lens, anterior chamber, posterior chamber and fundus.

### 2.9 Clinical pathology

The blood samples were collected via abdominal aorta after anaesthetization with pentobarbital sodium (i.p., 60 mg/kg) on the scheduled necropsy days of dosing phase and recovery phase. Hematological examination was performed using ADVIA 2120i hematology analyzer (Germany) and CS-5100 automatic coagulaiton analyzer (Japan) (Table 1). The clinical chemistry examination was assessed using Roche cobas 6000/c501 automatic biochemistry analyzer (Switzerland) (Table 1).

**TABLE 1:** Parameters evaluated in hematology, clinical chemistry and urinalysis.

Hematology	Clinical chemistry	Urinalysis
Red blood cell count (RBC)	Alkaline phosphate (ALP)	Urinary clarity (CLA)
Hemoglobin (HGB)	Alanine aminotransferase (ALT)	Urinary color (COL)
Hematocrit (HCT)	Aspartate aminotransferase (AST)	Glucose (GLU)
Mean corpuscular volume (MCV)	Creatine kinase (CK)	Bilirubin (BIL)
Mean corpuscular hemoglobin (MCH)	Gamma glutamyltransferase (GGT)	Ketones (KET)
Mean corpuscular hemoglobin concentration (MCHC)	Lactate dehydrogenase (LDH)	Specific gravity (SG)
Reticulocyte count (RET)	Urea (UREA)	Occult blood (BLO)
Reticulocyte percentage (RET%)	Creatinine (CREA)	pH value (pH)
White blood cell count (WBC)	Total protein (TP)	Protein (PRO)
Neutrophil (NEU) absolute count and percentage	Albumin (ALB)	Urobilinogen (URO)
Lymphocyte (LYM) absolute count and percentage	Albumin/Globulin ratio (A/G)	Nitrites (NIT)
Monocyte (MONO) absolute count and percentage	Glucose (GLU)	White Blood Cell (WBC)
Eosinophil (EOS) absolute count and percentage	Total bilirubin (TBIL)	—
Basophil (BASO) absolute count and percentage	Cholesterol (CHOL)	—
Platelet count (PLT)	Triglycerides (TG)	—
Prothrombin time (PT)	Sodium (Na^+^)	—
Activated partial thromboplastin time (APTT)	Potassium (K^+^)	—
—	Chloride (Cl^−^)	—

Urine samples were directly collected from the metabolic cage at the end of dosing phase (Day 26) and at the end of recovery phase (Recovery Day 28), respectively. Urinalysis parameters in Table 1 were evaluated using CLINITEK Atlas automatic urine analyzer (Germany).

### 2.10 Necropsy

Complete gross necropsies were conducted on all surviving animals at the end of treatment (the day after the last dosing) and recovery periods. The following selected organs were weighed and evaluated in terms of organ/body or organ/brain weight ratios: brain, kidneys, adrenals, testes, ovaries, uterus, epididymides, thymus, spleen, heart and liver. Paired organs were weighed together. Organ-to-body weight ratio = Organ weight(g)/Body weight(g) × 100%, and Organ-to-brain weight ratio = Organ weight(g)/Brain weight(g) × 100%.

### 2.11 Histopathology

The tissues in the Table 2 were preserved in 10% neutral phosphate-buffered formalin. Bilateral eyes, optic nerves, testes, epididymides and Harderian glands were fixed in modified Davidson’s solution. For all the above tissues in all groups, sampling, paraffin embedding, sectioning and hematoxylin-eosin staining was performed, followed by histopathology examination microscopically with light microscopy.

**TABLE 2:** Organs or tissues for histopathology examination.

Organs	Organs	Organs
Brain (cerebrum, cerebellum, diencephalon, brain stem, olfactory bulb)	Gland, pituitary	Spinal cord (cervical, thoracic, lumbar)
Liver	Spleen	Gland, adrenal
Pancreas	Thymus	Heart
Artery, aorta	Kidney	Lungs
Trachea	Esophagus	Gland, thyroid
Gland, parathyroid	Stomach	Small intestine, duodenum
Small intestine, jejunum	Small intestine, ileum	Large intestine, cecum
Large intestine, colon	Large intestine, rectum	Urinary bladder
Gland, salivary, submandibular	Testes	Epididymides
Gland, prostate	Seminal vesicle	Ovaries
Uterus	Oviduct	Vagina
Gland, mammary (male and female)	Skin (inguinal)	Muscle, biceps femoris
Nerve, sciatic	Eyes	Nerve, optic
Gland, Harderian	Bone and bone marrow (femur, with knee joint surface, right)	Cervix
Main-stem bronchi	Body cavity, nasal	Site, application (tail)
Lymph nodes (cervical, mesenteric, inguinal)		

### 2.12 Handling of dead animals

The animals found dead were recorded for the observation or death time, and weighed followed by a complete postmortem examination to explore the cause of death. All required organs and tissue as listed in the Section 2.11 and other tissues with gross lesions were performed with histopathology examination. Animals found dead which could not be examined immediately were temporarily refrigerated.

### 2.13 Toxicokinetics

Blood samples from animals in LPM3480392 groups were collected at predose and 2 min, 30 min, 1 h and 4 h postdose of the 1^st^ dosing on Days 1 and 28; at 2 min and 4 h postdose of the 2^nd^ dosing on Days 1 and 28; at 2 min, 1 h and 4 h postdose the 3^rd^ dosing on Days 1 and 28. Animals in control group were sampled at predose and 2 min postdose of the 1^st^ dosing on Days 1 and 28. Approximately 0.36 mL of blood was collected from the jugular vein of surviving TK animals at each time point. Whole blood samples were anticoagulated with EDTA-K_2_ and centrifuged at 2°C–8°C and 1800 × g for 10 min. The harvested plasma was stored below −66°C before the analysis. LC-MS/MS methods (liquid chromatograph/mass spectrometer) were used to detect concentrations of LPM3480392 at each time point. TK parameters (AUC, C_max_, T_max_) were calculated by Phoenix WinNonlin (Pharsight) 8.2. The detection instruments include ultra-performance liquid chromatography (Waters, United States ACQUITY UPLC I -CLASS) and triple quadrupole tandem mass spectrometer (Waters, United States, Xevo TQ-S).

### 2.14 Statistical analysis

Quantitative parameters were described as means ± standard deviation (X ± SD). The parameters included body weight, food consumption, hematology, clinical chemistry, urinary specific gravity (only at the end of recovery phase), organ weight and ratios, *et al.* Qualitative data (binomial category, unordered multi-category and ordinal multi-category) of urinalysis (including urinary specific gravity at the end of dosing phase) were presented as observed counts (frequency). When sample size was less than three, raw data of that group were directly presented without statistical comparison.

Statistical analysis was performed between each LPM3480392 group and the control group. Levene’s test was used to analyse the variance homogeneity of quantitative data. In the case of homogeneity of variance (*p* > 0.05), they were evaluated using one-way analysis of variance (ANOVA); in the case of heterogeneity of variances (*p* ≤ 0.05), Kruskal-Wallis (K-W) H test was used. And if ANOVA was with significant difference (*p* ≤ 0.05), Dunnett’s *t*-test (Dunnett) was used for pairwise comparisons; if ANOVA was not significant (*p* > 0.05), the statistical analysis was completed. When K-W H test was significant (*p* ≤ 0.05), Mann-Whitney (M-W) *U* test was used for pairwise comparisons; if K-W H test was not significant (*p* > 0.05), the statistical analysis was completed. Ordinal multi-category data were analysed by K-W H test. In the case of finding significant difference (*p* ≤ 0.05), M-W *U* test was used for pairwise comparison. Binomial category data were analysed by Fisher’s exact probabilities test (EXACT). If there was significant difference (*p* ≤ 0.05), Fisher EXACT was used for pairwise comparisons. Analyses were performed according to sex at two-tailed probability level (*α* = 0.05). PRISTIMA 7.2.0 was used for statistical analysis of body weight, hematology, clinical chemistry, organ weight and ratios. Stata/IC 15.0 for Windows was adopted for the statistical analysis of the rest data.

## 3 Results

### 3.1 Dose formulation analysis

For LPM3480392 dose formulations at 0.02 mg/mL, 0.04 mg/mL and 0.08 mg/mL prepared before the first dosing, the mean measured concentrations were 0.021 mg/mL, 0.041 mg/mL and 0.078 mg/mL, respectively. The RE% between measured value and nominal value were 5.0%, 2.5% and −2.5%, respectively. For LPM3480392 dose formulations at 0.02 mg/mL, 0.04 mg/mL and 0.08 mg/mL prepared before the last dosing, the mean measured concentrations were 0.019 mg/mL, 0.039 mg/mL and 0.078 mg/mL, respectively. The RE% between measured value and nominal value were −5.0%, −2.5% and −2.5%, respectively. No LPM3480392 injection was detected in control formulations.

### 3.2 Mortality

Two rats (1 male and 1 female, 2/30 animals) at 2.4 mg/kg/day were found dead on Days 3 and 15, respectively. They showed decreased activity and/or increased muscle tone, sternal recumbent posture after dosing, with no drug-related abnormalities in gross necropsy or histopathology.

### 3.3 Clinical observation

The rats in 0.6, 1.2 and 2.4 mg/kg/day groups displayed mild to severe increased activity, starting from Days 7, 4 and 1 during the dosing phase, respectively. And the animals in 2.4 mg/kg/day showed decreased activity (18 males, 8 females, 26/36 animals) only a few time points during the dosing phase. Muscle tone was also increased in 1.2 mg/kg/day group (17 males, 16 females, 33/38 animals) during the early stage of dosing phase in the first 5 days, and 2.4 mg/kg/day group (18 males, 18 females, 36/36 animals) during the mostly complete dosing phase, following by the gradually decreased incidence with time.

The rats in 2.4 mg/kg/day group occasionally showed hunchback (1 male, 1/36 animals), piloerection (1 male, 1/36 animals) and deep respiration or rale (4 males, 4/36 animals). During the middle and late stage of dosing phase, sporadic individual rats in 1.2 and 2.4 mg/kg/day groups had fester at the application site of tail or broken tail, with no drug-related abnormalities in histopathology examination, which was maybe caused by mechanical stimulation of long-term operation.

LPM3480392-treated rats showed good general health, normal spontaneous activity, with no other toxic reaction during the recovery phase.

### 3.4 Body weight

During the dosing phase, body weight gains were dose-dependently decreased in males from Day 16 to Day 27 at 0.6 mg/kg/day, and from Day 6 to Day 27 at 1.2 and 2.4 mg/kg/day. Interestingly, during the recovery phase, at the four time points, body weight gains were deeply reduced in the three LPM3480392 groups (Table 3).

**TABLE 3:** Effects of LPM3480392 on body weight (g) and mean change rate (%) in male rats.

Time points	Control	0.6 mg/kg/day		1.2 mg/kg/day		2.4 mg/kg/day	
	Body weight	Body weight	Change rate	Body weight	Change rate	Body weight	Change rate
Prior to 1st dosing	211.8 ± 13.7	213.2 ± 11.1	0.66	213.2 ± 9.4	0.66	213.9 ± 12.9	0.99
Day 6	243.9 ± 13.6	247.1 ± 9.6	1.31	243.6 ± 12.3	−0.12	234.0 ± 21.8	−4.06
Day 9	262.7 ± 16.1	268.8 ± 11.6	2.32	247.3 ± 10.6^*^	−5.86	249.9 ± 31.9	−4.87
Day 13	290.8 ± 18.3	292.6 ± 13.5	0.62	281.5 ± 15.7	−3.20	272.4 ± 27.9^*^	−6.33
Day 16	307.3 ± 18.4	300.8 ± 12.5	−2.12	297.1 ± 19.6	−3.32	281.1 ± 31.2	−8.53
Day 20	322.1 ± 19.5	321.4 ± 15.8	−0.22	313.4 ± 22.6	−2.70	297.6 ± 36.8	−7.61
Day 23	333.7 ± 19.8	332.6 ± 17.3	−0.33	325.9 ± 21.5	−2.34	299.3 ± 36.1^*^	−10.31
Day 27	350.6 ± 21.7	349.9 ± 20.1	−0.20	339.3 ± 23.2	−3.22	315.4 ± 36.4^*^	−10.04
Recovery day 5	386.5 ± 16.8	367.7 ± 23.5	−4.86	361.2 ± 14.2	−6.55	329.2 ± 48.5	−14.83
Recovery day 12	414.7 ± 18.8	395.5 ± 24.5	−4.63	388.0 ± 14.5	−6.44	365.9 ± 50.0	−11.77
Recovery day 19	438.8 ± 21.3	415.6 ± 25.4	−5.29	416.4 ± 19.3	−5.10	386.7 ± 51.8	−11.87
Recovery day 26	461.6 ± 22.6	442.1 ± 24.4	−4.22	444.3 ± 23.9	−3.75	413.2 ± 56.3	−10.49

* Note:Statistical significance was noted in the difference of mean value when compared with the control (*p* ≤ 0.05). The data for body weight were presented as mean ± SD. N = 15 in dosing phase and *n* = 5 in recovery phase (4M004 was found dead on Day 15).

Mean change rate= (Mean value of dose groups-Mean value of control group)/(Mean value of control group)×100%.

However, in females, body weight gains were increased at every time point during the dosing phase, while the increased degree of body weight gains seem to be less during the recovery phase (Table 4).

**TABLE 4:** Effects of LPM3480392 on body weight (g) and mean change rate (%) in female rats.

Time points	Control	0.6 mg/kg/day		1.2 mg/kg/day		2.4 mg/kg/day	
	Body weight	Body weight	Change rate	Body weight	Change rate	Body weight	Change rate
Prior to 1st dosing	178.1 ± 10.4	176.9 ± 7.3	−0.67	175.3 ± 7.7	−1.57	179.1 ± 8.3	0.56
Day 6	185.4 ± 9.5	194.0 ± 11.4	4.64	194.9 ± 10.5	5.12	194.7 ± 11.9	5.02
Day 9	190.4 ± 9.2	201.1 ± 13.7	5.62	200.1 ± 12.1	5.09	198.8 ± 14.7	4.41
Day 13	195.7 ± 10.0	209.5 ± 13.4 ^*^	7.05	215.3 ± 11.3 ^*^	10.02	211.2 ± 13.0 ^*^	7.92
Day 16	201.9 ± 10.2	214.4 ± 13.2 ^*^	6.19	218.9 ± 12.3 ^*^	8.42	209.1 ± 13.2	3.57
Day 20	209.7 ± 11.3	224.2 ± 15.1 ^*^	6.91	221.5 ± 14.8	5.63	220.8 ± 15.5	5.29
Day 23	215.9 ± 14.2	228.1 ± 15.3	5.65	232.4 ± 16.4 ^*^	7.64	221.7 ± 19.8	2.69
Day 27	218.6 ± 15.0	236.2 ± 15.3 ^*^	8.05	237.2 ± 15.4 ^*^	8.51	230.9 ± 19.3	5.63
Recovery day 5	229.8 ± 17.4	243.5 ± 9.3	5.96	237.7 ± 13.0	3.44	230.1 ± 19.1	0.13
Recovery day 12	236.5 ± 17.1	251.6 ± 10.2	6.38	244.8 ± 13.2	3.51	241.1 ± 21.0	1.95
Recovery day 19	252.0 ± 20.9	258.8 ± 12.5	2.70	259.9 ± 16.5	3.13	253.1 ± 25.8	0.44
Recovery day 26	257.5 ± 24.1	265.5 ± 15.7	3.11	261.8 ± 22.7	1.67	261.1 ± 25.5	1.40

* Note:Statistical significance was noted in the difference of mean value when compared with the control (*p* ≤ 0.05). The data for body weight were presented as mean ± SD. N = 15 in dosing phase and *n* = 5 in recovery phase (4F014 was found dead on Day 3).

Mean change rate= (Mean value of dose groups-Mean value of control group)/(Mean value of control group) ×100%.

### 3.5 Food consumption

During the dosing phase, at every time points, food consumption in males was dose-dependently reduced. Especially, from Day 10 to Day 17, food consumption was deeply decreased compared with other time points. And, during the recovery phase, decreased food consumption obtained the partial recovery (Table 5).

**TABLE 5:** Effects of LPM3480392 on food consumption (g/animal/cage/day) and mean change rate (%) in male rats.

Time points	Control	0.6 mg/kg/day		1.2 mg/kg/day		2.4 mg/kg/day	
	Food consumption	Food consumption	Change rate	Food consumption	Change rate	Food consumption	Change rate
Day 3	20.3 ± 2.5	19.7 ± 0.1	−2.96	19.2 ± 0.9	−5.42	17.8 ± 0.8	−12.32
Day 7	22.7 ± 0.4	20.8 ± 0.6	−8.37	20.5 ± 0.7	−9.69	20.0 ± 2.3	−11.89
Day 10	27.9 ± 0.8	23.0 ± 1.6 ^*^	−17.56	20.4 ± 1.8 ^*^	−26.88	20.1 ± 1.9 ^*^	−27.96
Day 14	25.4 ± 1.8	21.0 ± 1.3	−17.32	20.2 ± 0.7	−20.47	17.3 ± 5.2	−31.89
Day 17	23.8 ± 0.3	21.1 ± 1.9	−11.34	20.5 ± 1.7 ^*^	−13.87	20.1 ± 0.7 ^*^	−15.55
Day 21	23.8 ± 1.1	21.6 ± 0.8	−9.24	23.2 ± 1.2	−2.52	23.2 ± 1.1	−2.52
Day 24	22.4 ± 0.5	20.6 ± 1.0	−8.04	22.2 ± 1.1	−0.89	22.1 ± 3.0	−1.34
Day 28	24.3 ± 1.4	22.9 ± 1.2	−5.76	22.5 ± 1.3	−7.41	22.5 ± 1.9	−7.41
Recovery day 20	25.1	25.2	0.40	24.5	−2.39	24.0	−4.38
Recovery day 27	22.1	22.3	0.90	22.6	2.26	23.8	7.69

* Note:Statistical significance was noted in the group difference of mean value when compared with the control (*p* ≤ 0.05).

The data for food consumption were presented as mean ± SD. N = 3 in dosing phase and *n* = 1 in recovery phase (n = number of cages).

Mean change rate= (Mean value of dose groups-Mean value of control group)/(Mean value of control group) ×100%.

However, in females, during the dosing phase or the recovery phase, the food consumption was decreased at some time points while increased at the other time points, which did not present obvious pattern (Table 6).

**TABLE 6:** Effects of LPM3480392 on food consumption (g/animal/cage/day) and mean change rate (%) in female rats.

Time points	Control	0.6 mg/kg/day		1.2 mg/kg/day		2.4 mg/kg/day	
	Food consumption	Food consumption	Change rate	Food consumption	Change rate	Food consumption	Change rate
Day 3	14.8 ± 1.8	12.6 ± 1.3	−14.86	14.9 ± 1.1	0.68	13.0 ± 2.1	−12.16
Day 7	15.2 ± 0.5	15.4 ± 1.1	1.32	15.0 ± 1.3	−1.32	15.8 ± 1.9	3.95
Day 10	16.4 ± 0.9	15.9 ± 1.1	−3.05	15.2 ± 0.6	−7.32	15.1 ± 0.8	−7.93
Day 14	19.0 ± 0.2	16.0 ± 0.5 ^*^	−15.79	15.7 ± 0.9 ^*^	−17.37	17.5 ± 1.0	−7.89
Day 17	14.4 ± 1.0	15.8 ± 1.0	9.72	16.6 ± 0.2	15.28	15.1 ± 1.0	4.86
Day 21	16.0 ± 0.7	15.7 ± 0.7	−1.88	15.8 ± 1.4	−1.25	17.5 ± 1.3	9.38
Day 24	15.9 ± 2.4	14.9 ± 1.4	−6.29	16.6 ± 1.7	4.40	15.6 ± 1.2	−1.89
Day 28	16.5 ± 1.6	16.4 ± 0.9	−0.61	16.4 ± 0.8	−0.61	16.7 ± 0.8	1.21
Recovery day 20	16.8	15.9	−5.36	15.1	−10.12	16.4	−2.38
Recovery day 27	15.8	13.3	−15.82	16.7	5.70	15.0	−5.06

* Note:Statistical significance was noted in the group difference of mean value when compared with the control (*p* ≤ 0.05).

The data for food consumption were presented as mean ± SD. N = 3 in dosing phase and *n* = 1 in recovery phase (n = number of cages).

Mean change rate= (Mean value of dose groups-Mean value of control group)/(Mean value of control group)×100%.

### 3.6 Ophthalmic examination

At the end of dosing phase and recovery phase, both genders in 2.4 mg/kg/day group were observed with clear dioptric media and optic disk. The diameter of retinal artery was even. Veins were without apparent dilation. No abnormalities were noted in retina, optic disk, conjunctiva, cornea, iris, lens, anterior chamber or posterior chamber.

### 3.7 Hematology

At the end of dosing phase, for the males, 2.4 mg/kg/day of LPM3480392 induced the significantly increased RET/RET% and the significantly decreased EOS/EOS% and MONO, and 1.2 mg/kg/day significantly increased RET/RET% and decreased ESO. At the end of recovery phase, 2.4 mg/kg/day significantly increased RET% (Table 7).

**TABLE 7:** Effects of LPM3480392 on hematology of male rats.

Parameters	Control group	0.6 mg/kg/day	1.2 mg/kg/day	2.4 mg/kg/day
WBC (10^9^/L)				
End of dosing phase	5.49 ± 1.47	5.19 ± 1.38	4.56 ± 0.55	4.24 ± 0.98
End of recovery phase	5.06 ± 1.89	4.04 ± 1.12	4.50 ± 1.16	5.02 ± 0.40
MONO% (%)				
End of dosing phase	1.3 ± 0.3	1.3 ± 0.6	1.1 ± 0.3	0.9 ± 0.4
End of recovery phase	1.2 ± 0.4	1.0 ± 0.4	0.9 ± 0.3	1.1 ± 0.3
EOS% (%)				
End of dosing phase	1.02 ± 0.32	0.90 ± 0.42	0.77 ± 0.39	0.49 ± 0.29^*^
End of recovery phase	0.80 ± 0.49	1.14 ± 0.38	0.90 ± 0.12	0.66 ± 0.21
MONO (10^9^/L)				
End of dosing phase	0.07 ± 0.03	0.07 ± 0.03	0.05 ± 0.02	0.04 ± 0.02^*^
End of recovery phase	0.05 ± 0.01	0.04 ± 0.02	0.04 ± 0.02	0.05 ± 0.02
EOS (10^9^/L)				
End of dosing phase	0.056 ± 0.023	0.042 ± 0.013	0.035 ± 0.016^*^	0.022 ± 0.014^*^
End of recovery phase	0.038 ± 0.022	0.046 ± 0.018	0.042 ± 0.013	0.032 ± 0.011
RBC (10^12^/L)				
End of dosing phase	7.38 ± 0.45	7.05 ± 0.32	7.06 ± 0.32	7.05 ± 0.37
End of recovery phase	8.09 ± 0.28	7.96 ± 0.24	7.99 ± 0.29	7.62 ± 0.37
RET % (%)				
End of dosing phase	3.57 ± 0.80	4.26 ± 0.77	4.70 ± 0.92^*^	5.51 ± 0.83^*^
End of recovery phase	1.69 ± 0.19	1.81 ± 0.30	1.65 ± 0.21	2.07 ± 0.11^*^
RET (10^12^/L)				
End of dosing phase	0.263 ± 0.055	0.300 ± 0.051	0.331 ± 0.062^*^	0.387 ± 0.053^*^
End of recovery phase	0.136 ± 0.014	0.144 ± 0.021	0.132 ± 0.017	0.157 ± 0.008

* Note:Statistical significance was noted, compared with the control (*p* ≤ 0.05). At the end of dosing phase, *n* = 10 (*n* = 9 in 2.4 mg/kg/day group). At the end of recovery phase, *n* = 5. The data were presented as means ± standard deviation (X ± SD).

At the end of dosing phase, for the females, the significantly decreased MONO% or MONO in 0.6, 1.2 and 2.4 mg/kg/day groups, the significantly decreased RBC in 1.2 and 2.4 mg/kg/day groups, the increased MCV in 1.2 mg/kg/day group and the decreased PLT in 2.4 mg/kg/day group, were observed. At the end of recovery phase, the significantly increased LYM%, and the significantly decreased NEU/NEU%, and MONO% were noted in all LPM3480392 groups (Table 8).

**TABLE 8:** Effects of LPM3480392 on hematology of female rats.

Parameters	Control group	0.6 mg/kg/day	1.2 mg/kg/day	2.4 mg/kg/day
WBC (10^9^/L)				
End of dosing phase	3.83 ± 1.43	4.84 ± 0.93	4.87 ± 1.28	3.15 ± 0.87
End of recovery phase	4.85 ± 2.29	4.03 ± 0.57	3.42 ± 0.78	3.60 ± 0.67
NEU% (%)				
End of dosing phase	16.7 ± 9.8	10.6 ± 5.5	11.1 ± 7.2	15.4 ± 4.6
End of recovery phase	26.9 ± 6.3	10.6 ± 4.8^*^	10.6 ± 2.5^*^	12.8 ± 2.8^*^
LYM% (%)				
End of dosing phase	78.0 ± 11.4	86.1 ± 5.8	85.7 ± 7.2	81.1 ± 5.3
End of recovery phase	68.3 ± 6.3	85.8 ± 4.4^*^	85.9 ± 3.0^*^	83.7 ± 3.4^*^
MONO% (%)				
End of dosing phase	2.2 ± 1.5	1.1 ± 0.4^*^	0.9 ± 0.3^*^	1.0 ± 0.3^*^
End of recovery phase	2.3 ± 0.7	1.1 ± 0.4^*^	1.2 ± 0.4^*^	1.2 ± 0.3^*^
NEU (10^9^/L)				
End of dosing phase	0.55 ± 0.26	0.51 ± 0.26	0.50 ± 0.24	0.51 ± 0.29
End of recovery phase	1.29 ± 0.55	0.42 ± 0.17^*^	0.35 ± 0.08^*^	0.46 ± 0.09^*^
LYM (10^9^/L)				
End of dosing phase	3.11 ± 1.33	4.17 ± 0.90	4.21 ± 1.29	2.53 ± 0.59
End of recovery phase	3.33 ± 1.77	3.46 ± 0.53	2.95 ± 0.75	3.02 ± 0.62
MONO (10^9^/L)				
End of dosing phase	0.07 ± 0.02	0.05 ± 0.02	0.05 ± 0.02	0.03 ± 0.02^*^
End of recovery phase	0.11 ± 0.05	0.04 ± 0.02	0.04 ± 0.01	0.04 ± 0.01
RBC (10^12^/L)				
End of dosing phase	6.93 ± 0.31	6.90 ± 0.35	6.59 ± 0.26^*^	6.57 ± 0.30^*^
End of recovery phase	7.24 ± 0.56	7.56 ± 0.22	7.44 ± 0.33	7.45 ± 0.17
MCV (fL)				
End of dosing phase	56.2 ± 2.0	58.1 ± 1.5	58.7 ± 2.1^*^	58.1 ± 1.9
End of recovery phase	56.1 ± 1.9	56.4 ± 1.2	56.6 ± 1.8	56.8 ± 0.8
PLT (10^9^/L)				
End of dosing phase	1102 ± 129	1072 ± 103	1064 ± 104	946 ± 108^*^
End of recovery phase	962 ± 40	975 ± 144	975 ± 42	998 ± 45

* Note:Statistical significance was noted, compared with the control (*p* ≤ 0.05). At the end of dosing phase, *n* = 10. At the end of recovery phase, *n* = 5 (*n* = 4 in 2.4 mg/kg/day group). The data were presented as means ± standard deviation (X ± SD).

With the exception of the above changes, no significant differences were observed in other parameters in males or females treated with LPM3480392 at the end of dosing phase and recovery phase as compared with the control (*p* > 0.05).

### 3.8 Clinical chemistry

At the end of dosing phase, for the males, 0.6, 1.2 and 2.4 mg/kg/day induced the significantly decreased ALB and TP; 1.2 and 2.4 mg/kg/day significantly decreased TG; and 2.4 mg/kg/day significantly increased GLU. At the end of recovery phase, 1.2 and 2.4 mg/kg/day significantly increased A/G, and decreased GLU (Table 9).

**TABLE 9:** Effects of LPM3480392 on clinical chemistry of male rats.

Parameters	Control group	0.6 mg/kg/day	1.2 mg/kg/day	2.4 mg/kg/day
ALB(g/L)				
End of dosing phase	42.1 ± 2.0	39.8 ± 2.0^*^	38.4 ± 2.0^*^	38.0 ± 1.6^*^
End of recovery phase	37.7 ± 1.5	37.4 ± 0.4	36.6 ± 0.8	37.7 ± 0.7
TP(g/L)				
End of dosing phase	55.9 ± 2.5	53.0 ± 2.3^*^	51.3 ± 2.3^*^	51.0 ± 1.4^*^
End of recovery phase	58.1 ± 3.2	55.2 ± 1.7	54.5 ± 0.6	54.6 ± 1.7
TG(mmol/L)				
End of dosing phase	0.33 ± 0.14	0.21 ± 0.06	0.19 ± 0.05^*^	0.17 ± 0.05^*^
End of recovery phase	0.33 ± 0.18	0.26 ± 0.16	0.33 ± 0.12	0.31 ± 0.12
GLU(mmol/L)				
End of dosing phase	5.92 ± 0.70	5.90 ± 0.48	6.39 ± 0.32	6.76 ± 0.56^*^
End of recovery phase	8.03 ± 0.76	7.26 ± 0.55	6.64 ± 0.99^*^	6.53 ± 0.83^*^
A/G				
End of dosing phase	3.08 ± 0.27	3.02 ± 0.34	3.03 ± 0.37	2.94 ± 0.30
End of recovery phase	1.85 ± 0.11	2.10 ± 0.18	2.05 ± 0.08^*^	2.25 ± 0.27^*^

* Note:Statistical significance was noted compared with the control (*p* ≤ 0.05). At the end of dosing phase, *n* = 10 (*n* = 9 in 2.4 mg/kg/day group). At the end of recovery phase, *n* = 5. The data were presented as means ± standard deviation (X ± SD).

At the end of dosing phase, for the females, 0.6, 1.2 and 2.4 mg/kg/day induced the significantly decreased ALB, TP, TG, A/G; 1.2 and 2.4 mg/kg/day significantly decreased TBIL; and 2.4 mg/kg/day significantly decreased CREA, and increased AST, GLU and ALP (Table 10).

**TABLE 10:** Effects of LPM3480392 on clinical chemistry of female rats.

Parameters	Control group	0.6 mg/kg/day	1.2 mg/kg/day	2.4 mg/kg/day
ALB(g/L)				
End of dosing phase	48.1 ± 3.1	43.3 ± 2.6^*^	40.1 ± 2.0^*^	39.9 ± 1.9^*^
End of recovery phase	47.2 ± 2.9	46.4 ± 2.4	47.0 ± 3.5	45.5 ± 2.1
TP(g/L)				
End of dosing phase	59.5 ± 3.1	56.7 ± 2.2^*^	55.2 ± 2.1^*^	55.2 ± 1.4^*^
End of recovery phase	63.8 ± 4.1	63.8 ± 2.6	65.1 ± 4.0	63.4 ± 3.3
AST(U/L)				
End of dosing phase	112.7 ± 9.4	126.9 ± 16.0	122.5 ± 16.8	132.4 ± 15.4^*^
End of recovery phase	109.9 ± 17.3	103.0 ± 8.5	95.9 ± 16.3	105.9 ± 15.9
ALT(U/L)				
End of dosing phase	27.0 ± 6.1	24.9 ± 4.2	28.0 ± 8.0	28.7 ± 4.2
End of recovery phase	32.2 ± 6.6	29.0 ± 6.6	29.2 ± 2.7	34.0 ± 7.0
TBIL(μmol/L)				
End of dosing phase	1.3 ± 0.4	1.0 ± 0.5	0.7 ± 0.2^*^	0.7 ± 0.2^*^
End of recovery phase	1.6 ± 0.4	1.1 ± 0.5	1.2 ± 0.3	0.9 ± 0.6
TG(mmol/L)				
End of dosing phase	0.32 ± 0.18	0.19 ± 0.05^*^	0.19 ± 0.03^*^	0.16 ± 0.03^*^
End of recovery phase	0.28 ± 0.08	0.28 ± 0.08	0.27 ± 0.03	0.30 ± 0.06
CREA(μmol/L)				
End of dosing phase	29.4 ± 3.5	27.9 ± 2.4	29.4 ± 2.4	24.4 ± 1.5^*^
End of recovery phase	34.6 ± 9.4	36.2 ± 3.7	35.2 ± 1.3	32.5 ± 3.1
GLU(mmol/L)				
End of dosing phase	5.87 ± 0.72	6.20 ± 0.43	6.44 ± 0.43	6.66 ± 0.45^*^
End of recovery phase	7.58 ± 1.77	7.01 ± 0.37	6.80 ± 0.80	6.61 ± 0.24
ALP(U/L)				
End of dosing phase	79.8 ± 25.2	87.4 ± 25.6	95.9 ± 26.3	124.1 ± 41.1^*^
End of recovery phase	46.9 ± 12.6	60.5 ± 13.8	51.5 ± 17.8	59.2 ± 16.1
A/G				
End of dosing phase	4.31 ± 0.76	3.25 ± 0.35^*^	2.70 ± 0.42^*^	2.64 ± 0.37^*^
End of recovery phase	2.88 ± 0.41	2.68 ± 0.12	2.63 ± 0.37	2.55 ± 0.13

* Note:Statistical significance was noted in the difference of mean value when compared with the control (*p* ≤ 0.05). At the end of dosing phase, *n* = 10. At the end of recovery phase, *n* = 5 (*n* = 4 in 2.4 mg/kg/day group). The data were presented as means ± standard deviation (X ± SD).

With the exception of the above, no significant differences were observed in other clinical chemistry parameters for males or females treated with LPM3480392 at the end of dosing phase and recovery phase as compared with the control (*p* > 0.05).

### 3.9 Urinalysis

For the male rats, the grade distribution of NIT at 1.2 and 2.4 mg/kg/day, and grade distribution of KET at 2.4 mg/kg/day were significantly decreased compared with the concurrent control (*p* ≤ 0.05) at the end of dosing phase (Table 11).

**TABLE 11:** Effects of LPM3480392 on urinalysis.

Parameters	Detection time grade	Control group	0.6 mg/kg/day	1.2 mg/kg/day	2.4 mg/kg/day
KET (mmol/L) of males	End of dosing phase	—	—	—	*
	—	1	1	1	5
	1.5	4	3	3	0
	3.9	0	1	1	0
	N	5	5	5	5
	End of recovery phase	—	—	—	—
	—	2	2	2	4
	1.5	2	2	2	1
	3.9	1	1	1	0
	N	5	5	5	5
NIT of males	End of dosing phase	—	—	*	*
	—	0	3	4	4
	+	5	2	1	1
	N	5	5	5	5
	End of recovery phase	—	—	—	—
	—	2	2	4	5
	+	3	3	1	0
	N	5	5	5	5
pH of females	End of dosing phase	—	—	—	*
	7.0	4	1	1	0
	7.5	1	3	3	1
	8.5	0	1	1	3
	N	5	5	5	4
	End of recovery phase	—	*	—	—
	6.0	0	0	1	0
	6.5	2	0	0	2
	7.0	3	0	2	2
	7.5	0	4	1	0
	8.0	0	1	1	0
	N	5	5	5	4

Note: *Statistical significance was noted in the difference of frequency distribution when compared with the control (*p* ≤ 0.05).

For the female rats, the grade distribution of pH at 2.4 mg/kg/day at end of dosing phase, and at 0.6 mg/kg/day at end of recovery phase were significantly increased compared with the concurrent control (*p* ≤ 0.05) (Table 11).

With the exception of the above, there were no significant differences in urinary parameters for the males or females treated with LPM3480392 at the end of dosing phase and recovery phase as compared with the control (*p* > 0.05).

### 3.10 Organ weight and ratios

For the male rats, at the end of dosing phase, the significantly increased organ-to-body weight ratio of heart at 0.6, 1.2 and 2.4 mg/kg/day, the significantly decreased absolute weight of liver and adrenal gland at 1.2 and 2.4 mg/kg/day, and the significantly decreased absolute weight of epididymides and the organ-to-brain weight ratio of liver at 2.4 mg/kg/day were observed. At the end of recovery phase, only the significantly increased absolute weight of thymus was noted in 0.6 mg/kg/day group (Table 12).

**TABLE 12:** Effects of LPM3480392 on organ weight and ratios of male rats.

Parameters	Control group	0.6 mg/kg/day	1.2 mg/kg/day	2.4 mg/kg/day
Terminal BW (g)				
End of dosing phase	328.1 ± 23.3	322.9 ± 16.9	308.5 ± 22.5	296.6 ± 24.2^*^
End of recovery phase	392.6 ± 94.3	425.2 ± 26.4	424.6 ± 23.0	395.7 ± 53.7
Organ weight (g)				
Liver				
End of dosing phase	8.496 ± 0.972	8.125 ± 0.587	7.643 ± 0.801^*^	7.348 ± 0.497^*^
End of recovery phase	11.409 ± 1.582	9.943 ± 1.685	10.423 ± 1.077	9.451 ± 1.196
Adrenal gland				
End of dosing phase	0.061 ± 0.007	0.052 ± 0.011	0.052 ± 0.007^*^	0.052 ± 0.004^*^
End of recovery phase	0.059 ± 0.008	0.057 ± 0.008	0.060 ± 0.010	0.055 ± 0.009
Epididymis				
End of dosing phase	1.034 ± 0.085	0.977 ± 0.089	1.020 ± 0.040	0.904 ± 0.104^*^
End of recovery phase	1.297 ± 0.132	1.432 ± 0.140	1.351 ± 0.094	1.239 ± 0.114
Heart				
End of dosing phase	1.171 ± 0.122	1.289 ± 0.102	1.199 ± 0.140	1.172 ± 0.071
End of recovery phase	1.381 ± 0.068	1.431 ± 0.112	1.483 ± 0.081	1.360 ± 0.262
Brain				
End of dosing phase	2.063 ± 0.101	2.094 ± 0.050	2.015 ± 0.137	2.031 ± 0.066
End of recovery phase	2.157 ± 0.123	2.262 ± 0.071	2.177 ± 0.033	2.185 ± 0.147
Spleen				
End of dosing phase	0.750 ± 0.136	0.741 ± 0.128	0.703 ± 0.103	0.686 ± 0.129
End of recovery phase	0.827 ± 0.088	0.774 ± 0.108	0.814 ± 0.089	0.775 ± 0.172
Thymus				
End of dosing phase	0.438 ± 0.095	0.477 ± 0.062	0.425 ± 0.115	0.394 ± 0.075
End of recovery phase	0.348 ± 0.065	0.470 ± 0.076^*^	0.393 ± 0.042	0.386 ± 0.038
Organ-to-body weight ratio (%)				
Liver				
End of dosing phase	2.584 ± 0.138	2.516 ± 0.108	2.473 ± 0.100	2.487 ± 0.195
End of recovery phase	3.078 ± 0.992	2.327 ± 0.250	2.451 ± 0.149	2.391 ± 0.064
Adrenal gland				
End of dosing phase	0.019 ± 0.002	0.016 ± 0.003	0.017 ± 0.002	0.018 ± 0.002
End of recovery phase	0.016 ± 0.006	0.013 ± 0.002	0.014 ± 0.003	0.014 ± 0.002
Epididymis				
End of dosing phase	0.317 ± 0.033	0.303 ± 0.028	0.332 ± 0.030	0.305 ± 0.028
End of recovery phase	0.362 ± 0.170	0.338 ± 0.036	0.318 ± 0.010	0.315 ± 0.026
Heart				
End of dosing phase	0.357 ± 0.021	0.399 ± 0.028^*^	0.388 ± 0.025^*^	0.397 ± 0.037^*^
End of recovery phase	0.380 ± 0.148	0.337 ± 0.020	0.349 ± 0.017	0.342 ± 0.024
Brain				
End of dosing phase	0.631 ± 0.049	0.650 ± 0.035	0.655 ± 0.040	0.689 ± 0.061
End of recovery phase	0.594 ± 0.237	0.534 ± 0.040	0.514 ± 0.031	0.559 ± 0.076
Spleen				
End of dosing phase	0.228 ± 0.032	0.229 ± 0.030	0.227 ± 0.026	0.231 ± 0.033
End of recovery phase	0.220 ± 0.052	0.182 ± 0.018	0.192 ± 0.022	0.194 ± 0.023
Thymus				
End of dosing phase	0.133 ± 0.026	0.147 ± 0.016	0.136 ± 0.028	0.132 ± 0.019
End of recovery phase	0.096 ± 0.042	0.110 ± 0.016	0.093 ± 0.013	0.099 ± 0.013
Organ-to-brain weight ratio (%)				
Liver				
End of dosing phase	412.43 ± 48.33	388.28 ± 30.51	378.95 ± 25.83	361.66 ± 16.84^*^
End of recovery phase	532.65 ± 98.74	441.01 ± 85.94	478.76 ± 49.84	432.91 ± 49.50
Adrenal gland				
End of dosing phase	2.98 ± 0.44	2.50 ± 0.58	2.59 ± 0.37	2.59 ± 0.24
End of recovery phase	2.77 ± 0.50	2.52 ± 0.37	2.74 ± 0.50	2.55 ± 0.51
Epididymis				
End of dosing phase	50.24 ± 4.82	46.67 ± 4.12	50.88 ± 4.79	44.58 ± 5.64^*^
End of recovery phase	60.10 ± 4.25	63.42 ± 6.91	62.05 ± 4.34	56.82 ± 5.54
Heart				
End of dosing phase	56.85 ± 5.91	61.61 ± 5.76	59.48 ± 5.54	57.77 ± 3.83
End of recovery phase	64.07 ± 2.42	63.29 ± 4.73	68.08 ± 3.40	62.11 ± 10.37
Spleen				
End of dosing phase	36.35 ± 6.20	35.42 ± 6.22	35.01 ± 5.62	33.71 ± 5.76
End of recovery phase	38.49 ± 5.02	34.27 ± 5.33	37.41 ± 4.09	35.31 ± 6.72
Thymus				
End of dosing phase	21.17 ± 4.21	22.74 ± 2.77	20.87 ± 4.42	19.36 ± 3.42
End of recovery phase	16.09 ± 2.52	20.83 ± 3.66	18.03 ± 1.83	17.77 ± 2.29

* Note:Statistical significance was noted in the difference of mean value when compared with the control (*p* ≤ 0.05).

For the female rats, at the end of dosing phase, the significantly decreased organ-to-body weight ratio of brain at 0.6 and 1.2 mg/kg/day, and the significantly decreased organ-to-body weight ratio of adrenal gland at 1.2 mg/kg/day were observed (Table 13).

**TABLE 13:** Effects of LPM3480392 on organ weight and ratios of female rats.

Parameters	Control group	0.6 mg/kg/day	1.2 mg/kg/day	2.4 mg/kg/day
Terminal BW (g)				
End of dosing phase	205.3 ± 12.7	213.5 ± 15.3	217.8 ± 15.0	208.0 ± 14.6
End of recovery phase	241.7 ± 18.5	251.2 ± 9.7	248.4 ± 12.5	244.2 ± 24.2
Organ weight (g)				
Kidney				
End of dosing phase	1.533 ± 0.142	1.501 ± 0.123	1.518 ± 0.127	1.549 ± 0.122
End of recovery phase	1.598 ± 0.251	1.626 ± 0.142	1.567 ± 0.165	1.554 ± 0.133
Brain				
End of dosing phase	1.944 ± 0.080	1.862 ± 0.102	1.916 ± 0.096	1.958 ± 0.105
End of recovery phase	1.953 ± 0.104	2.018 ± 0.046	1.990 ± 0.086	2.075 ± 0.093
Spleen				
End of dosing phase	0.623 ± 0.096	0.646 ± 0.159	0.637 ± 0.078	0.616 ± 0.144
End of recovery phase	0.554 ± 0.140	0.530 ± 0.085	0.574 ± 0.056	0.502 ± 0.064
Thymus				
End of dosing phase	0.369 ± 0.111	0.402 ± 0.053	0.380 ± 0.046	0.339 ± 0.075
End of recovery phase	0.365 ± 0.091	0.308 ± 0.055	0.297 ± 0.056	0.331 ± 0.135
Organ-to-body weight ratio (%)				
Kidney				
End of dosing phase	0.747 ± 0.049	0.704 ± 0.037	0.697 ± 0.044	0.746 ± 0.049
End of recovery phase	0.658 ± 0.062	0.647 ± 0.043	0.630 ± 0.037	0.637 ± 0.024
Brain				
End of dosing phase	0.949 ± 0.052	0.875 ± 0.063^*^	0.881 ± 0.039^*^	0.943 ± 0.055
End of recovery phase	0.813 ± 0.087	0.804 ± 0.031	0.802 ± 0.031	0.856 ± 0.093
Spleen				
End of dosing phase	0.302 ± 0.035	0.301 ± 0.065	0.294 ± 0.042	0.294 ± 0.049
End of recovery phase	0.228 ± 0.048	0.210 ± 0.030	0.231 ± 0.016	0.206 ± 0.027
Thymus				
End of dosing phase	0.178 ± 0.045	0.189 ± 0.028	0.175 ± 0.026	0.162 ± 0.031
End of recovery phase	0.150 ± 0.030	0.122 ± 0.019	0.120 ± 0.023	0.135 ± 0.047
Organ-to-brain weight ratio (%)				
Kidney				
End of dosing phase	78.85 ± 6.43	80.71 ± 6.29	79.14 ± 3.61	79.23 ± 6.43
End of recovery phase	81.91 ± 12.18	80.51 ± 6.21	78.65 ± 6.18	75.09 ± 8.25
Adrenal gland				
End of dosing phase	3.13 ± 0.29	3.21 ± 0.59	2.89 ± 0.37	2.88 ± 0.24
End of recovery phase	2.87 ± 0.32	2.91 ± 0.20	2.68 ± 0.38	2.71 ± 0.42
Spleen				
End of dosing phase	32.01 ± 4.60	34.73 ± 8.54	33.29 ± 3.96	31.37 ± 6.57
End of recovery phase	28.27 ± 6.30	26.23 ± 4.03	28.83 ± 2.34	24.11 ± 2.01
Thymus				
End of dosing phase	18.92 ± 5.59	21.61 ± 3.02	19.90 ± 2.88	17.23 ± 3.33
End of recovery phase	18.65 ± 4.15	15.21 ± 2.43	14.89 ± 2.48	16.14 ± 7.16

* Note:Statistical significance was noted in the difference of mean value when compared with the control (*p* ≤ 0.05).

With the exception of above changes, no significant differences were noted in the absolute weight and ratios of other organs of LPM3480392-treated males or females at the end of dosing phase and recovery phase as compared with the control (*p* > 0.05).

### 3.11 Gross necropsy

There were no LPM3480392-related abnormalities in the size, morphology, texture or color of main organs such as brain, heart, liver, spleen, kidney, adrenal gland, gastrointestinal tract, uterus, testes, epididymides at the end of dosing phase and recovery phase.

### 3.12 Histopathology

**FIGURE 2:**
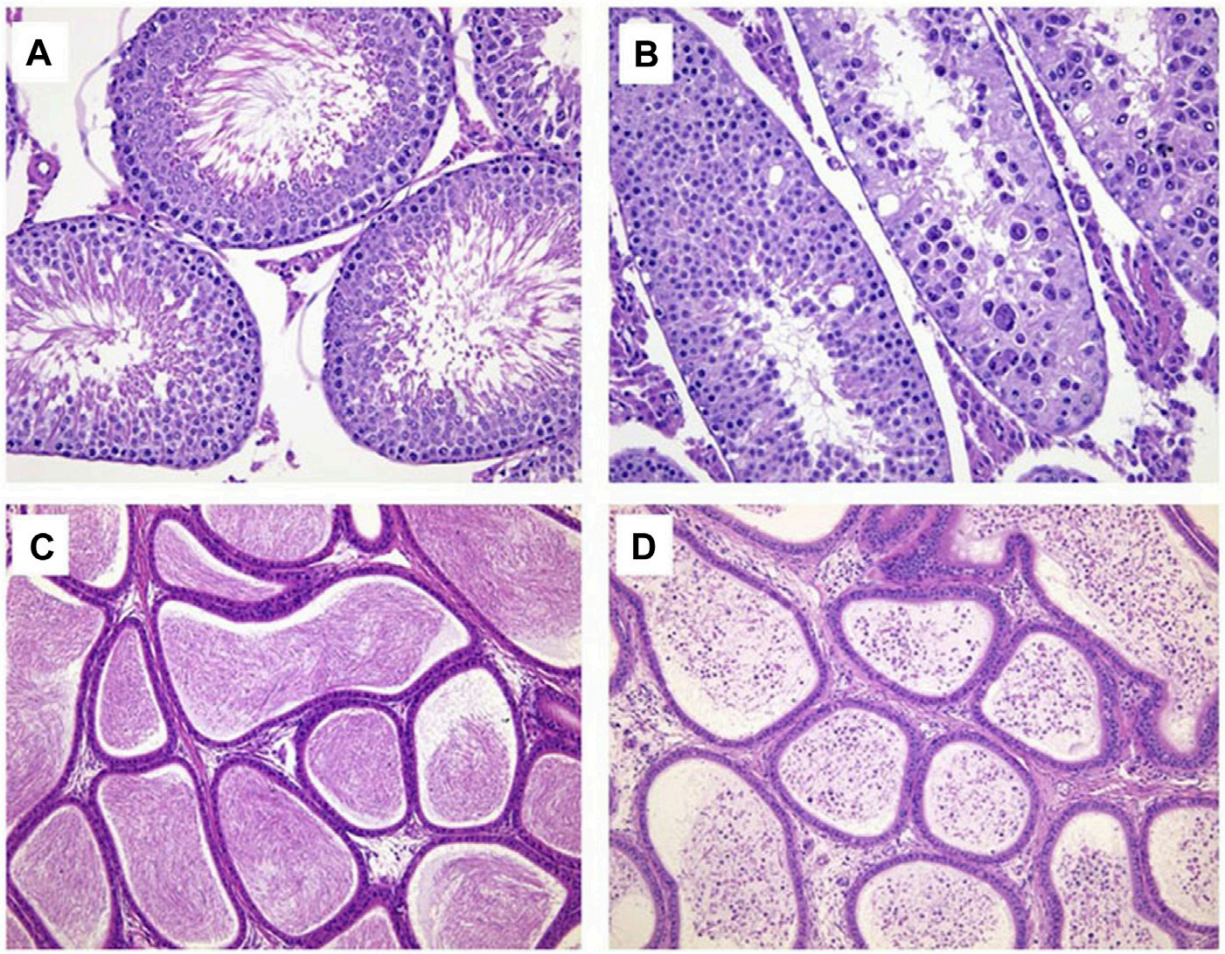
Hematoxylin and eosin-stained histologic sections for testis (× 200) and epididymis (× 100). **(A)** Normal testis in control group; **(B)** Tubule degeneration of testis at 2.4 mg/kg/day. **(C)** Normal epididymis in control group; **(D)** Cell debris and decreased sperm in the lumen of epididymis at 2.4 mg/kg/day.

Sporadic individual males at 2.4 mg/kg/day were observed with the mild tubular degeneration of the testis (1/9), the marked decreased sperm in the lumen and the minimal to moderate cell debris in the lumen of the epididymis (1/9) at the end of dosing phase. The above changes were not identified at the end of recovery phase (Table 14; Figure 2).

**TABLE 14:** LPM3480392-related microscopic findings at the end of dosing phase.

Group		Control		0.6 mg/kg/day		1.2 mg/kg/day		2.4 mg/kg/day	
Microscopic		M	F	M	F	M	F	M	F
Testis	Number examined	10	—	10	—	10	—	9	—
Degeneration, tubule	Grade: 2	0	—	0	—	0	—	1	—
	Total Incidence	0	—	0	—	0	—	1	—
Epididymis	Number examined	10	—	10	—	10	—	9	—
Sperm, decreased, lumen	Grade: 4	0	—	0	—	0	—	1	—
	Total Incidence	0	—	0	—	0	—	1	—
Cell debris, lumen	Grade: 1	0	—	0	—	0	—	1	—
	Grade: 3	0	—	0	—	0	—	1	—
	Total Incidence	0	—	0	—	0	—	2	—

Note: Grade: 1 = minimal, 2 = mild, 3 = moderate, 4 = marked, 5 = severe. — = not applicable.

With the exception of the above, no microscopic changes were observed in brain, heart, liver, spleen, lungs, kidney, adrenal gland, thymus, lymph node, and gastrointestinal tract of LPM3480392 -treated rats at the end of dosing phase and recovery phase.

### 3.13 Toxicokinetics

The concomitant toxicokinetics study was conducted for LPM3480392. Over the dose range from 0.6–2.4 mg/kg/day, the mean AUC_0–12h_ of LPM3480392 in plasma had no apparent gender difference, and was increased generally in a dose-proportional manner on Day 1 and Day 28. No accumulation of LPM3480392 was noted after the 28-day repeated dosing.

**FIGURE 3:**
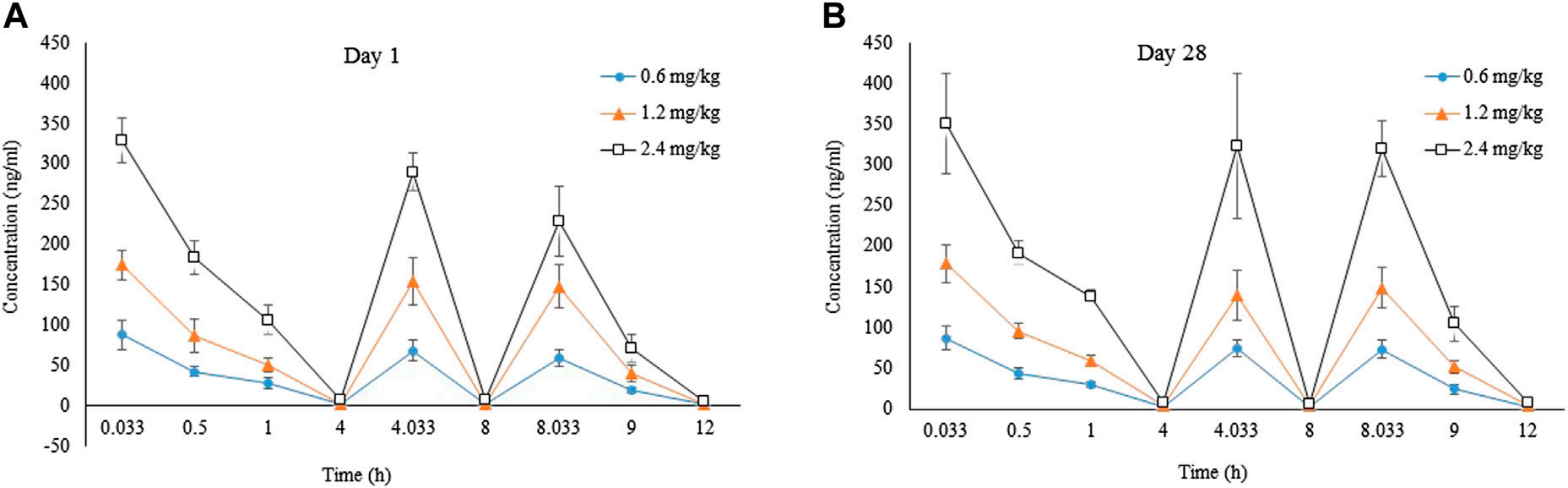
Tokicokinetics profile for LPM3480392. Mean plasma LPM4870059 concentration-time curve in rats (F + M) intravenously administered with LPM3480392 on Day 1 **(A)** and Day 28 **(B)**.

On Day 1, mean AUC_0–12h_ were 315, 663 and 1250 h ng/mL for rats (female + male) in each group, with the between-group ratio of 1:2.1:4.0. On Day 28, mean AUC_0–12h_ were 349, 680 and 1490 h ng/mL for rats (female + male) in each group, with the between-group ratio of 1:1.9:4.3. Compared with Day 28, mean AUC_0–12h_ ratios of LPM3480392 in plasma were 1.1, 1.0 and 1.2 for rats (female + male) in each group on Day 1, respectively, (Table 15; Figure 3).

**TABLE 15:** TK parameters for plasma LPM3480392 after intravenous administration in rats.

Dosage (mg/kg/day) (dosing day)	Sex	AUC_0–12h_ (h•ng/mL)	C_2min_ (ng/mL) (The 1st dosing)	C_2min_ (ng/mL) (The 2nd dosing)	C_2min_ (ng/mL) (The 3rd dosing)
0.6 (1st)	F	319.0 ± 56.0	78.4 ± 10.9	66.2 ± 12.4	55.5 ± 8.94
	M	310.0 ± 35.6	98.1 ± 21.3	70.5 ± 14.7	64.7 ± 10.8
	F + M	315.0 ± 43.7	88.3 ± 18.8	68.4 ± 12.8	60.1 ± 10.4
1.2 (1st)	F	719.0 ± 122.0	181.0 ± 24.8	169.0 ± 26.2	157.0 ± 25.5
	M	607.0 ± 83.4	170.0 ± 10.7	142.0 ± 28.9	139.0 ± 29.2
	F + M	663.0 ± 114.0	175.0 ± 18.6	155.0 ± 29.4	148.0 ± 27.0
2.4 (1st)	F	1310.0 ± 57.9	331.0 ± 25.1	292.0 ± 33.6	234.0 ± 52.1
	M	1190.0 ± 87.1	328.0 ± 33.8	289.0 ± 10.5	225.0 ± 40.9
	F + M	1250.0 ± 93.6	330.0 ± 27.6	290.0 ± 23.1	229.0 ± 43.6
0.6 (28th)	F	331.0 ± 48.3	90.1 ± 8.8	73.0 ± 12.6	68.5 ± 11.8
	M	366.0 ± 14.7	84.3 ± 20.2	77.9 ± 8.2	79.4 ± 7.61
	F + M	349.0 ± 38.2	87.2 ± 14.7	75.4 ± 10.2	73.9 ± 10.9
1.2 (28th)	F	735.0 ± 63.4	187.0 ± 20.0	167.0 ± 14.1	166.0 ± 16.1
	M	625.0 ± 28.9	171.0 ± 26.2	113.0 ± 9.1	131.0 ± 18.7
	F + M	680.0 ± 74.5	179.0 ± 23.2	140.0 ± 31.0	149.0 ± 24.6
2.4 (28th)	F	1580.0 ± 129.0	356.0 ± 81.9	387.0 ± 54.9	323.0 ± 20.7
	M	1400.0 ± 168.0	346.0 ± 46.0	259.0 ± 68.3	317.0 ± 47.9
	F + M	1490.0 ± 169.0	351.0 ± 61.7	323.0 ± 89.2	320.0 ± 34.2

Note: TK, toxicokinetics; AUC_0–12h_, Area under the concentration-time curve from 0 to 12 h; C_2min_, drug concentration at minute 2; F, female; M, mlae.

## 4 Discussion

LPM3480392, a full MOR biased agonist, demonstrates *in vitro* biased agonism without β-arrestin-2 recruitment activity, which induces potent antinociceptive effect with reduced respiratory suppression (Yang et al., 2022). In the present study, we evaluated the subacute toxicity of LPM3480392 in rats. Two rats (1 male and 1 female) at 2.4 mg/kg/day were found dead on Days 3 and 15, respectively. They showed decreased activity and/or increased muscle tone, sternal recumbent posture after dosing, with no drug-related gross necropsy or histopathology changes. There is no clear death cause found.

Opiates-induced locomotor hyperactivity in rodents involves activation of the mesolimbic dopamine system and serves as one behavioural consequence of enhanced mesolimbic dopamine signalling (Botz-Zapp et al., 2021; Liu et al., 2021; Paul et al., 2021; Santos et al., 2023). However, at a higher dose, morphine induced the decreased locomotor activity(Paul et al., 2021). Opiates, such as morphine can induce dose-dependent muscular activity (rigidity), which was explained by decreased striatal dopaminergic activity (Wand et al., 1973; Havemann et al., 1982). Traditional opiates can induce severe adverse effects (SAE) such as respiratory suppression by activating both Gi and β-arrestin signaling pathway (Bohn et al., 1999; Bohn et al., 2000; Raehal et al., 2005; Del Vecchio et al., 2017; Zhuang et al., 2022). In rodent studies, TRV130, LPM3480392 and other G-protein-biased MOR agonists produce less respiratory depressions than traditional opioids (Bossert et al., 2020; Yang et al., 2022). In this study, during the dosing phase, the rats in 0.6, 1.2 and 2.4 mg/kg/day groups mostly displayed increased activity while 2.4 mg/kg/day also induced the decreased activity only a few time points. LPM3480392 at 1.2 and 2.4 mg/kg/day increased muscle tone. In addition, the sporadic individual rats occasionally had deep respiration or rale, hunched back and piloerection in 2.4 mg/kg/day group. Only the rats (4/36) in 2.4 mg/kg/day group were observed with deep respiration or rale. LPM3480392-treated rats showed no activity abnormalities during the recovery phase.

The opiates-induced body weight decrease could be due to increased energy expenditure (Levine et al., 1988). And, the body weight decrease induced by opiates withdrawal may be mediated by the activation of stress-related brain circuits (Bobzean et al., 2019; Jimenez-Romero et al., 2020). LPM3480392 treatment resulted in the decreased body weight gains and food consumption in males during the dosing phase, which are typically observed with opiate drugs (Mitzelfelt et al., 2011). Especially, body weight gains were pronounced reduced in the three LPM3480392 groups during the recovery phase, compared with the dosing phase, which is a typical predictive factor of opiates withdrawal (Glick et al., 1975; Pinelli and Trivulzio, 1997; Binsack et al., 2006; Mitzelfelt et al., 2011; Le et al., 2014; Townsend et al., 2022). However, though the decreased food consumption in females were observed in several time points during dosing phase and recovery phase, the body weight gains were increased. No obvious effects on body weight gain in females were observed in other opiates studies (Bobzean et al., 2019; Jimenez-Romero et al., 2020; Trevena, Inc, 2020; Taylor et al., 2023), such as the 4-week continuous IV infusion toxicity study of TRV130 (Trevena, Inc, 2020) and prolonged morphine administration (Taylor et al., 2023). The sex difference of the effects on body weight especially after drug withdrawal might reflect sex difference in cellular activation (phosphorylated CREB) in the GABAergic neurons of the ventral tegmental area (Bobzean et al., 2019).

In this study, we regret that we did not carefully observe the withdrawal behaviour. However, we have conducted the spontaneous withdrawal test in rats in 2019 at WCFP (data not shown in this manuscript). The rats were intravenously injected with LPM3480392 injection from 0.3 to 1.8 mg/kg/day for 30 consecutive days. Physical dependence was induced by LPM3480392 injection in rats after the spontaneous withdrawal. Increased scores of the withdrawal behaviours such as wet dog shaking, writhing, chew, teeth chattering and eyelid ptosis were noted in LPM3480392 injection groups after the drug withdrawal. These withdrawal behaviours had a gradually recovery trend, which had reserved to normal on Recovery day 6 for rats administered with LPM3480392 injection. After the withdrawal, body weight of male rats in LPM3480392 injection group significantly decreased and then recovered to normal within the withdrawal recovery phase. There were no apparent abnormalities in body weights of female rats in LPM3480392 injection groups during dosing and recovery phases. The effects of LPM3480392 on body weight in males or females in the spontaneous withdrawal test are similar to those in the subacute toxicity study in rats.

Opiates have been conventionally considered immunosuppressive. The µ-opioid receptor is essential to the immunosuppressive effects observed after morphine administration (Plein and Rittner, 2018; Franchi et al., 2019; Ackerman et al., 2021). Not all opiates yield immunosuppression (Franchi et al., 2019; Ackerman et al., 2021). Morphine and fentanyl have been found to impair the function of macrophages, natural killer cells and T-cells in animal studies (Plein and Rittner, 2018; Franchi et al., 2019). However, The immunosuppressive effects has not been observed with oxycodone or buprenorphine (Plein and Rittner, 2018; Franchi et al., 2019; Ackerman et al., 2021). Though the impact of G-protein vs. β-arrestin activation in immunosuppression has never been explored, buprenorphine does not recruit β-arrestin to the receptor and has been reported to be devoid of immunosuppressive properties (Franchi et al., 2019). In our study, no histopathological changes were observed in spleen, thymus and lymph nodes in both males and females after LPM3480392 treatment. Also, there are no significant decreases in the absolute/relative organ weight of spleen and thymus in males or females. In the male rats, the mildly lowered MONO and EOS/EOS% was observed at the end of dosing phase. In the female rats, the mildly decreased MONO/MONO% at the end of dosing phase; and the mildly increased LYM%, and the decreased NEU/NEU% and MONO% were observed at the end of recovery phase. However, the absolute WBC were not significantly changed in males or females at the end of dosing phase or recovery phase. All the changes about WBC were considered not toxicologically significant because of evidence of the in-house background range, and absence of dose dependence and time dependence, and the related microscopic correlation. Importantly, in the concomitant toxicokinetics study, the mean AUC_0–12h_ and C_max_ of LPM3480392 were increased generally in a dose-proportional manner on Day 1 and Day 28. No accumulation of LPM3480392 was noted after the 28-day repeated dosing. After every LPM3480392 treatment, the drug was eliminated fast in the body (Table 15; Figure 3). Therefore, LPM3480392 cannot produce long-lasting hematologic issues. Based on the above results, LPM3480392 did not display obvious immunosuppressive effects maybe because of this compound’s full µ-opioid receptor bias.

A few LPM3480392-related minor changes in other haematology results were observed. At the end of dosing phase, only small magnitude of increases in RET/RET% in the male rats were noted without the abnormalities in peripheral blood erythroid series or histopathology of bone marrow, and the changes of MCV, RBC and PLT in the female rats were with small magnitude (less than 5%, 6% and 15%, respectively). All the changes were considered not toxicologically significant because of their small magnitude of changes, evidence of reversibility, and absence of microscopic correlation.

The effects of decreased food intake on a series of toxicological parameters in rats have been widely understood, including haematology, clinical chemistry, urinalysis, organ weight, and histopathology (Levin et al., 1993; Moriyama et al., 2008). The reduced TG, ALB, TG and GLU were attributable to the nutritional deficiency secondary to food intake reduction (Levin et al., 1993; Moriyama et al., 2008). In this study, in all LPM3480392 groups, the decreased TG (<49%), GLU (<20%), ALB (<10%) and TP (<10%) in the males, and decreased ALB (<18%) and TP (<18%) in the females were observed, which could be related to the decreased food consumption resulted from LPM3480392 treatment. The decreased TBIL in females could be induced by LPM3480392, which aren’t generally a cause for concern as a liver function index. In the female rats, the increased AST and GLU and decreased CREA at all LPM3480392 groups were observed with small magnitude (<18%). All the changes in clinical chemistry parameters were considered not toxicologically significant, because of their small magnitude of changes, evidence of reversibility, absence of microscopic correlation, and no direct target organs toxicity.

In urinalysis, the decreased grade distribution of NIT and KET in the males of 1.2 and/or 2.4 mg/kg/day groups, and the increased grade distribution of pH in the females of 2.4 mg/kg/day group were observed at the end of dosing phase; and PH changes in 0.6 mg/kg/day group were also noted at the end of recovery phase. The above changes had no mostly toxicologically significant, as no abnormalities were noted in serum renal function related parameters or histopathology of kidney and urinary system.

It is generally accepted that µ-opioid agonists such as morphine and fentanyl activate the hypothalamus-pituitary-adrenal (HPA) axis in rodents (Tanabe and Cafruny, 1958; Franchi et al., 2007), and chronic administration of morphine or TRV130 produces adrenal cortical hypertrophy(Tanabe and Cafruny, 1958; Trevena, Inc, 2020;). However, in our study, the decreased absolute weight in adrenal gland of males, and the decreased organ-to-body weight ratio of adrenal gland of females were noted in LPM3480392 groups, but there were no histopathology changes. We did not determine if a full µ-opioid receptor bias produce this difference compared with other opiates. At the end of dosing phase, the increase in organ-to-body weight ratio of heart but not heart weight was observed in all LPM3480392-treated male rats, which is due to the reduced body weight gain. The slightly decreased absolute weight or organ-to-brain weight ratio of liver in LPM3480392-treated male rats were observed, while no significant live-related clinical chemistry and histopathology changes. The decreased organ-to-body weight ratio of brain of females were presented, but no absolute brain weight changes at the end of dosing phase, which was related to the increased body weight gain. At the end of recovery phase, the increased thymus weight of males in 0.6 mg/kg/day group was noted without toxicological significance, because of no changes at the end of dosing phase, no dose-dependency or microscopic abnormalities. Therefore, the changes of heart, liver, brain, thymus and adrenal gland were considered not toxicologically significant, because of their small magnitude of changes, evidence of reversibility, no dose-dependency, and absence of histopathology changes.

At the end of recovery phase, the decreased absolute weight and organ-to-brain weight ratio of epididymides were observed in 2.4 mg/kg/day group. Sporadic individual males at 2.4 mg/kg/day were observed with the mild tubular degeneration of the testis, the marked decreased sperm in the lumen and the minimal to moderate cell debris in the lumen of the epididymis. The changes of testis and epididymis were maybe target organ toxicity for LPM3480392. However, the above microscopic changes of testis and epididymis were recovered at the end of recovery phase.

The toxicokinetics study shown that typical linear kinetics characteristics were presented on Day 1 and Day 28 after LPM3480392 administration, and the mean AUC_0–12h_ was increased generally in a dose-proportional manner. No accumulation of LPM3480392 was noted after the 28-day repeated dosing. The highest drug exposure was observed at 2.4 mg/kg/day, which partly explained the toxicity, including the unscheduled death, the clinical signs of increased activity, increased muscle tone, sternal recumbent posture, deep respiration; microscopic changes of tubular degeneration of the testis, and decreased sperm in the lumen and cell debris in the lumen of the epididymis.

In this study, there are several limitations: (1) We missed the observation time points for the opiates withdrawal such as the decreased body weight and food consumption, and wet-dog shaking during the first days of recovery phase. (2) Morphine can induce mydriasis in rats (Klemfuss et al., 1979; Adler et al., 1981). However, we did not observe if LPM3480392 induced the mydriasis in the ophthalmic examination during the dosing and recovery phases. We will carry out the related experiments in order to observe the opiates withdrawal behaviours and mydriasis.

In summary, LPM3480392 presents weak/no immunosuppression and the decreased adrenal gland weight, which is different from other opiates. The main reason may be that LPM3480392 is a full MOR biased agonist. Further experiments need to be warranted in order to study the effects of LPM3480392 on immune system and HPA axis.

## Data Availability

The original contributions presented in the study are included in the article/Supplementary material, further inquiries can be directed to the corresponding authors.
